# Correction: Vatani, A., *et al*. Prediction of Standard Enthalpy of Formation by a QSPR Model. *Int. J. Mol. Sci.* 2007, *8*, 407–432.

**DOI:** 10.3390/ijms10020723

**Published:** 2009-02-24

**Authors:** Ali Vatani, Mehdi Mehrpooya, Farhad Gharagheizi

**Affiliations:** Department of Chemical Engineering, Faculty of Engineering, University of Tehran, P.O. Box: 11365-4563, Tehran, Iran

We found an error in our paper published in the *Int. J. Mol. Sci.* recently [1]: on page 409, the Equation (2) should be printed as:

$$\Delta H_{f}^{˚}=50.1688-80.52012nSK+53.64546SCBO-169.21889nO-174.75477nF-266.57659nHM$$

We apologize for any inconvenience caused to the readers.
